# Quantitative proteomic Analysis Reveals up-regulation of caveolin-1 in FOXP3-overexpressed human gastric cancer cells

**DOI:** 10.1038/s41598-017-14453-2

**Published:** 2017-10-31

**Authors:** Duyi Pan, Jing Gao, Xiaoqing Zeng, Guifen Ma, Na Li, Xiaoquan Huang, Xuanling Du, Qing Miao, Jingjing Lian, Lili Xu, Hu Zhou, Shiyao Chen

**Affiliations:** 1 Department of Gastroenterology, Zhongshan Hospital, Fudan University, Shanghai, China; 2 Department of Radiotherapy, Zhongshan Hospital, Fudan University, Shanghai, China; 3 Department of Analytical Chemistry and CAS Key Laboratory of Receptor Research, Shanghai Institute of Materia Medica, Chinese Academy of Sciences, Shanghai, China; 4 University of Chinese Academy of Sciences, Beijing, 100049 China

## Abstract

Forkhead box protein 3 (FOXP3) is implicated in tumor progression and prognosis in various types of tumor cells. We have recently reported that FOXP3 inhibited proliferation of gastric cancer (GC) cells through activating the apoptotic signaling pathway. In this study, we found that over-expression of FOXP3 inhibited GC cell migration, invasion and proliferation. Then, the label-free quantitative proteomic approach was employed to further investigating the down-stream proteins regulated by FOXP3, resulting in a total of 3,978 proteins quantified, including 186 significantly changed proteins. Caveolin-1 (CAV1), as a main constituent protein of caveolae, was one of those changed proteins up-regulated in FOXP3-overexpressed GC cells, moreover, it was assigned as one of the node proteins in the protein-protein interaction network and the key protein involved in focal adhesion pathway by bioinformatics analysis. Further biological experiments confirmed that FOXP3 directly bound to the promoter regions of CAV1 to positively regulate CAV1 transcription in GC cells. In summary, our study suggested that FOXP3 can be considered as a tumor suppressor in GC via positively regulating CAV1 through transcriptional activation, and this FOXP3-CAV1 transcriptional regulation axis may play an important role in inhibiting invasion and metastasis of GC cells. Data are available via ProteomeXchange under identifier PXD007725.

## Introduction

Gastric cancer (GC) is a common malignant tumor worldwide^1^. Although the incidence of GC is declining in most of developed countries, it is still one of the most common causes of cancer-related death in many Asian countries. In China, gastric cancer is the third and second cause of death for both men and women and the main cancers in rural areas. With an incidence of 42.92/10^5^ and a mortality of 29.93/10^5^ ^2^, gastric cancer is still a great threat to people’s health in China.

The gene of FOXP3 is located on the short arm of the X chromosome at Xp.11.23^3^. FOXP3 is a transcription factor that is necessary for induction of the immunosuppressive functions of regulatory T cells (Tregs), and it is a well-known marker of Tregs^4^. Recently, FOXP3 is reported to be expressed in various kinds of tumor cell including colorectal cancer^5^, melanoma^6^, non-small cell lung cancer^7^ and many other cancer cell lines^8^. Suh *et al*. found that tumoral FOXP3 expression is associated with favorable clinicopathological variables in gastric adenocarcinoma, and FOXP3 is associated with the Hippo pathway proteins, Lats2 and YAP expression^9^. Hao *et al*. demonstrated that FOXP3 was dominantly expressed in GC cells, and FOXP3 can act as a negative regulator of NF-κB activity to play a tumor suppressor role by reducing GC cell metastasis^10^.

In our previous studies, we have demonstrated that FOXP3 suppressed the GC cells proliferation by induction of GC cells apoptosis, and positive histological staining of FOXP3 in GC cells indicated better outcome^11,12^. The experimental results of FOXP3 in our previous studies were consistent with other findings in breast cancer and gastric cancer^9,13^. However, there is a huge difference for clinic treatment between early gastric cancer and metastatic gastric cancer. Hence, our study aimed to investigate the effects of FOXP3 on GC cell adhesion, migration and invasion and the mechanisms behind.

Label-free proteomic analysis is a recently emerged, efficient, powerful, and cost-effective approach for comparing multiple samples from different cells/tissues. In our previous studies, label-free proteomic analysis was applied to analyzing clinic and animal samples, such as endometrial tissues in proliferative and receptive phases^14^, omental adipose tissues from patients with gestational diabetes mellitus^15^, colonic biopsies from endoscopy^16^, and mouse corneal tissues^17^. Proteomic strategies were also employed to investigating the function of FOXP3. Rudra *et al*. purified FOXP3 complexes and explored their composition with mass spectrometry-based proteomics and identified 361 associated proteins, ~30% of which are transcription-related^18^. Kubach *et al*. reported a 2-dimensional gel electrophoresis (2D PAGE)-based proteomic work, and galectin-10 was identified as a novel marker and essential for CD4+ CD25+ FOXP3+ regulatory T cells anergy and suppressive function^19^.

In this study, overexpression of FOXP3 significantly inhibited cell migration, invasion and proliferation. Then label-free proteomic experiments were performed to analyze the FOXP3-overexpressed AGS cells and vector cells, resulting in a total of 3978 proteins identified, of which 186 proteins were significantly changed (fold change >1.5, students’ t test p value <0.01) between these two types of cells. The expression of CAV1 was significantly increased in AGS cells and played a central role in the protein-protein interaction networks constructed by these significantly changed proteins. Moreover, the results from KEGG pathway analyses enlightened the significant changes in the focal adhesion pathway, and CAV1 played an important biological role in this pathway. ChIP-PCR and luciferase assay were applied for biological validation, confirming that CAV1 was a direct transcription target of FOXP3. These results indicated that CAV1 may play an important role as the downstream transcriptional target of FOXP3 in regulation the adhesion, migration and invasion functions of GC cells.

## Materials and Methods

### Cell culture

Human GC cells (AGS) were obtained from the Cell Culture Center of Institute of Biochemistry and Cell Biolgy, Chinese Academy of Science (Shanghai, China). The AGS cells were maintained in Rosewell Park Memorial Institute (RPMI) 1640 medium (Hyclone, USA) supplemented with 10% fetal bovine serum (Invitrogen, USA) at 37 °C in a humidified atmosphere of 5% CO_2_.

### Cell transfection

For cell transfection, AGS cells were grown to 70 to 80% confluence in six well plates. Transfection was performed using lentivirus with a multiplicity of infection (MOI) of 50 (Shanghai GenePharma Co.,Ltd. China). The lentiviral vector containing FOXP3-specific gene was cotransfected with packaging vector pVSV-G into 293 T cells. The supernatant which contains virus was collected and added onto AGS cells 48 h after cotransfection. Cells stably expressing FOXP3 were selected using 2.5 μg/mL puromycin. Overexpression of FOXP3 was determined by using western blot to quantify the level of FOXP3 protein. The FOXP3-overexpressing cells were named AF and the vector-transfected cells were named ANC.

### Protein trypsin digestion using the filter aided sample preparation (FASP) method

1 × 10^7^ of cells were washed with ice-cold PBS three times, then lysed in SDT buffer containing 4% SDS (m/w), 100 mM DTT, 100 mM Tris, pH 7.6. The lysate was incubated at 95 °C for 5 min and then centrifuged at 15,000 *g* for 10 min, and the supernatant was used for the proteomics sample preparation. 100 μg of protein sample was digested using FASP method as previously described^20^. Each sample was mixed with 200 μL of UA buffer (8 M urea, 0.1 M Tris–HCl, pH = 8.5), loaded on a 10k Microcon filtration device (EMD Millipore, Billerica, MA, USA) and centrifuged at 14,000 *g* for 40 min at 20 °C. The concentrates were diluted in the device with 200 μL of UA solution and centrifuged again under the same conditions. The concentrate was then mixed with 100 μL of 50 mM indole acetic acid (IAA) in UA buffer and incubated for an additional 40 min at room temperature in darkness. After this, the IAA was removed by centrifugation at 14,000 *g* for 20 min. Next, the sample was diluted with 200 μL of UA buffer and centrifuged twice. Subsequently, 200 μL of 50 mM NH_4_HCO_3_ was added and the sample was centrifuged at 14,000 *g* for 40 min; this step was repeated twice. Finally, 100 μL of 5 0 mM NH_4_HCO_3_ and trypsin (1:50, enzyme to protein) were added to the sample, which was then incubated at 37 °C for 16 h. The tryptic peptide mixtures were collected for further analysis.

### LC-MS/MS analysis

The reverse phase high performance liquid chromatography (RP-HPLC) separation was achieved on the EASY-nLC1000 HPLC system (Thermo Fisher Scientific,Grand Island, NY, USA) using a self-packed column (75 μm × 150 mm; 3 μm ReproSil-Pur C18 beads, 120 Å, Dr. Maisch GmbH, Ammerbuch, Germany) at a flow rate of 300 nL/min using 240 min gradients. The MS data were acquired on an Orbitrap Elite Hybrid Ion Trap-Orbitrap Mass Spectrometer (Thermo Fisher scientific, Grand Island, NY, USA) platform in a data dependent strategy, selecting the top 20 CID fragmentation events based on the precursor abundance in the survey scan (350–1600 m/z). For accurate mass measurements, the lock-mass option was employed^21^. The full mass was scanned in the Orbitrap analyzer with R = 60,000 (defined at m/z 400), and the subsequent MS/MS analyses were performed with R = 15,000.

The MS data were analyzed using the software MaxQuant^22^ (http://maxquant.org/, version 1.5.1.0). Carbamidomethyl (C) was set as a fixed modification, and oxidation (M, +15.99492 Da) was set as a variable modification. Proteins were identified by searching the MS and MS/MS data for the peptides against a decoy version of the International Protein Index (IPI) human database (version 3.87, 91,491 protein sequences; European Bioinformatics Institute). Trypsin/P was selected as the digestive enzyme with two potential missed cleavages. Protein abundance was calculated according to the normalized spectral protein intensity (LFQ intensity). The MS proteomics data have been deposited into the ProteomeXchangeConsortium^23^ via the PRIDE partner repository with the dataset identifier PXD007725.

### Data analysis

Further analysis of the MaxQuant output protein quantitation data was performed using the Perseus software (version 1.5.0.15)^24^. First, hits to the reverse database, contaminants were eliminated. Then the LFQ intensities were logarithmized, imputed with random numbers from a normal distribution (width = 0.3, shift = 1.8), and sent to Student’s t-test analysis. Hierarchical clustering analysis was performed with the pheatmap package, which is based on the open-source statistical language R^25^, using Euclidean distance as the distance metric and complete method as the agglomeration method. A strict criterion including protein fold change >= 1.5 and p value of t-test analysis <= 0.01 was used for significant protein screening. The significant proteins were searched against the STRING database (version 10)^26^ for protein-protein interactions. STRING defines a metric called “confidence score” to define interaction confidence; we fetched all interactions which had a confidence score ≥ 0.7 (high confidence). The resulting interaction network had 64 nodes and 75 interactions. The network was visualized with Cytoscape software^27^ and protein regulation information was indicated as node color. Pathways enriched with significantly changing proteins were determined using a homemade pathway mapping tool based on the KEGG (Kyoto Encyclopedia of Genes and Genomes) pathway database^28^. The enrichment within a given pathway was assessed by the hypergeometric distribution.

### RNA isolation and quantitative real-time polymerase chain reaction (qRT-PCR)

Total RNA was isolated from transfected and non-transfected cells using a commercial RNA isolation kit (TaKaRa Biotechnology, Japan) according to the manufacturer’s instructions. RNA (2 μL) from each sample was reverse transcribed to cDNA. The total volume was 10 μL. The complementary DNA reverse transcribed from the RNA was amplified by a Taq polymerase using the following primers for the FOXP3 cDNA: 5′-AAGCAGCACTACATTGACCTGAAA-3′ (forward) and 5′-GGTCTCCCCAAGCATCACTC-3′ (reverse). CAV1: 5′-GGGCATTTACTTCGCCATT-3′ (forward) and 5′-TGGAATAGACACGGCTGATG-3′ (reverse); GAPDH was also amplified as a control, using the following primers: 5′-GCACCGTCAAGGCTGAGAAC-3′ (forward) and 5′-TGGTGAAGACGCCAGTGGA-3′ (reverse). The amplification reaction was initiated by denaturing DNA at 95 °C for 5 min, followed by 30 cycles of template denaturing at 94 °C for 1 min, primer annealing at 60 °C for 1 min, and primer extension at 72 °C for 1 min. The qRT-PCR was applied using an ABI PRISM 7500 System (Perkin-Elmer, USA).

### Western blot analysis

Protein expression in AGS cells was validated by western blotting. Cultured cells were collected and extracted using SDT buffer. The proteins were separated by molecular weight using 10% SDS-PAGE and then transferred to 0.22 μm polyvinyldifluoride membrane (PVDF) membrane (EMD Millipore, Billerica, MA, USA) at 100 V for 120 min. The blots were blocked with 5% BSA in Tris-buffered saline-Tween 20 (TBST buffer), followed by incubation with primary antibodies against GAPDH (Sigma aldrich, Saint Louis, MI), FOXP3(Abcam, Cambridge, United Kingdom) and CAV1 (Cell Signaling Technology, Danvers, MA, USA) overnight. The blots were washed three times with TBST and were incubated with the corresponding horseradish peroxidase conjugated secondary antibody (Santa cruz, USA) for 1 h. The signals were detected using an enhanced chemiluminescence (ECL) solution (Thermo Fisher scientific, Grand Island, NY, USA), and were captured by a gel imaging system (Amersham Imager 600; GE Healthcare, Waukesha, WI, USA). GAPDH protein was used as a loading control to normalize the Western blot data, and the relative values were calculated by the intensity of FOXP3 or CAV1 divided by the intensity of the loading control.

### *In vitro* wound-healing assay

The motility of AGS cells was assessed using a wound-healing assay. AF cells and ANC cells were seeded in six well plates at a density of 10^5^ cells/well in complete RPMI 1640. After incubation overnight to yield confluent monolayers for wounding, and washing the cells twice with PBS, 200 μL pipette tips were used to make wounds (4–5 parallel scratches/plate); subsequently, the cells were incubated with serum-free 1640 medium. Photographs were taken immediately (0 h) and 24 h after wounding from five randomly selected fields in each well. The distance migrated by the cell monolayer to close the wounded area during this time period was measured using ImageJ.

### Colony-formation assay

AF cells and ANC cells were seeded at an original density of 200 cells per 6 mm dish in a complete RPMI 1640 medium. After incubation at 37 °C for 7 days, adherent cells were washed twice and fixed with 4% paraformaldehyde for 30 min. They were then stained with methyl violet and washed twice with double distilled water. All cell colonies that were larger than 50 cells (2 mm in diameter) were counted in three separate dishes and the results expressed as mean ± SEM.

### Transwell invasion assay

For the invasion assay, Matrigel (Becton-Dickinson, Franklin Lakes, NJ, USA) was diluted in RPMI 1640 medium and 50 μL of the 0.5 mg Matrigel was evenly inoculated onto the upper chamber of a transwell membrane placed in a 24 well plate and allowed to form a gel at 37 °C. Subsequently, 2 × 10^5^ cells in RPMI 1640 containing 1% FBS were seeded on the upper chamber of the Matrigel-coated transwell filter (8 μm pore, BD Biosciences, USA). RPMI 1640 containing 10% fetal bovine serum was added to the lower chamber and incubated for 36 h at 37 °C. At the end of the incubation period, non-invasive cells were removed from the upper surface of the membrane by cotton wool. The cells attached to the lower chamber were fixed with 4% paraformaldehyde for 30 min, washed with ddH_2_O, and air dried. Cells on the opposite side of the chamber (transmigrated) were stained with 0.1% methyl violet for 30 min, washed, and viewed under an upright microscope (×400, NIKON). Cell migration was defined as the number of cells that remained on the membrane in five random fields.

### Chromatin immunoprecipitation-polymerase chain reaction (ChIP-PCR)

AGS cells at a concentration of 2 × 10^6^/mL were treated with 1% formaldehyde in medium for 10 min. 1.25 M glycine dissolved in PBS was added to a concentration of 0.125 M, and the sample was incubated for 5 min at room temperature to stop cross-linking reaction. Then cells were washed twice in cold PBS and then disrupted in 1% SDS lysis buffer. Then sonication was performed to keep the DNA fragments in an average length between 200 to 500 bp as verified by agarose gel electrophoresis. The chromatin was immunoprecipitated with antibodies (3 μL) directed against FOXP3, with equal amounts of IgG as negative controls. The same amount of chromatin without antibody incubation was used as the input control. The samples were incubated for 4 hours at 4 °C. The reaction mixtures were eluted following the manufacturer’s protocol. Co-precipitated DNAs were purified with phenol-chloroform, and detected by qRT-PCR.

### Dual-luciferase reporter assay

AGS cells at 80–90% confluence were transiently transfected with a series of luciferase reporter expression vectors together with the sh-FOXP3 plasmid or the sh-NC plasmid. 48 h later, the luciferase activity was measured using luciferase assay substrate. The luciferase activity was normalized to the Renilla luciferase activity. Data are presented as the relative mean ± SD in triplicates. *p < 0.05 vs DMSO.

## Results and Discussion

### Overexpression of FOXP3 inhibits cell migration, invasion and proliferation

**Figure 1:**
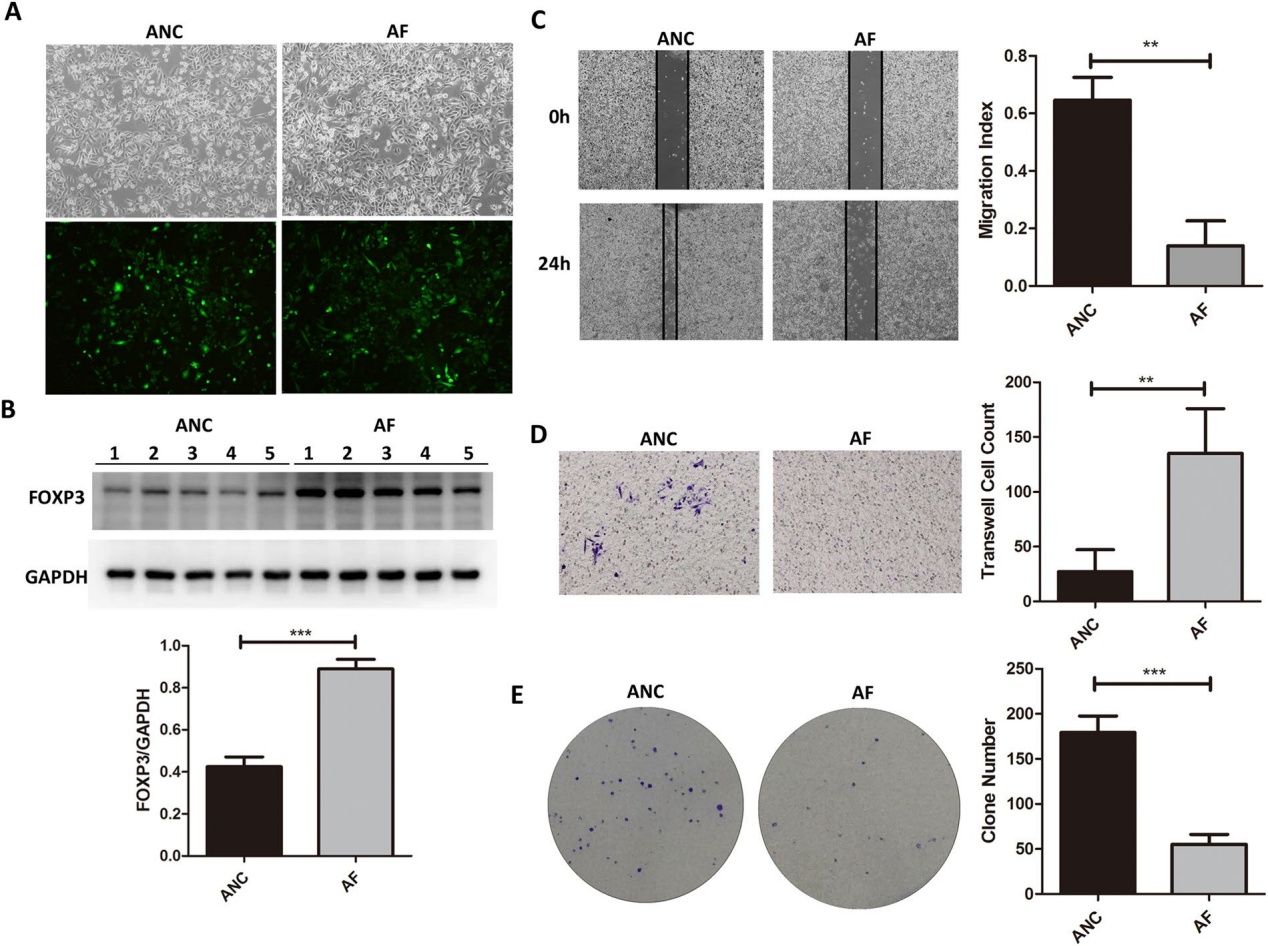
Overexpression of FOXP3 inhibited GC cell migration, invasion and proliferation. (**A**) overexpression of FOXP3 in GC cells by lentivirus transfection. (**B**) Western blot confirmed FOXP3 protein expression in ANC and AF cells. The cell motility(**C**), migration ability(**D**) and proliferation rate (**E**) was inhibited in AF cells. AF: FOXP3-overexpressing AGS cells; ANC: vector controls.

FOXP3 was over-expressed in GC cells with lentivirus transfection (AF cells), and the empty vector was also transfected into GC as control cells (ANC cells). As shown in Fig. 1A, cells transfected with FOXP3-containing lentivirus showed equal green fluorescence comparing with the vector-transfected cells. As expected, the protein expression level of FOXP3 significantly increased in AF cells (2.09 fold, t-test p value <  = 0.01) (Fig. 1B, Original Western Blot images shown in Supplementary Fig. 4). Then the cell migration capability was assessed and compared between AF and ANC cells. In the wound-healing assay, the open wound area (%) of AF was significantly greater than that of ANC (Fig. 1C), indicating that FOXP3 significantly inhibited GC cell motility. The migration of GC cells was also significantly inhibited in FOXP3-overexpressing groups in the transwell migration assay (Fig. 1D). Cells had migrated across the pore of the membrane from the upper chamber of the transwell to the lower chamber. The number of migrated cells was lower for AF when compared with ANC, suggesting that FOXP3 inhibited GC cell migration. Representative clones of AF and ANC were seeded in 6 cm dishes. FOXP3-overexpressing cells showed significant suppression of their ability to form colonies (p < 0.05) when compared with control cells (Fig. 1E). In summary, these results suggested that overexpression of FOXP3 leads to suppression of GC cell adhesion, migration and invasion.

### Label-free quantitative proteomic analysis of control GC cells and FOXP3-overexpressed GC cells

**Figure 2:**
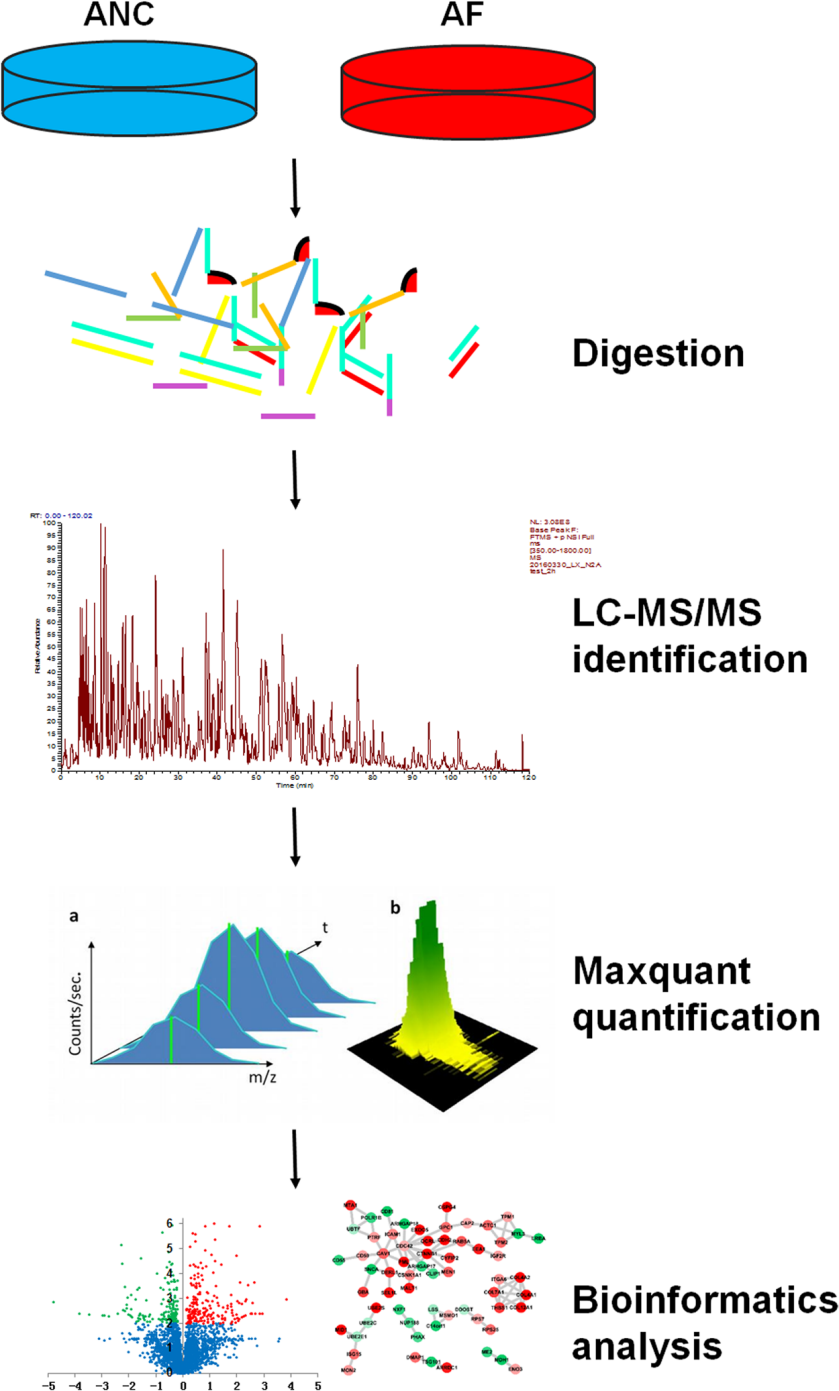
Workflow of the label-free quantitative proteomic experiments. Five biological replications of AF and ANC cell lines were involved for proteomics analyze. Proteins were extracted and digested by FASP method. The resulting peptides were analyzed by nano-LC−MS/MS, quantified with the label-free algorithm in MaxQuant software. The significant proteins were analyzed by KEGG and protein−protein interaction network analyses and sent for further functional investigation.

To investigate the down-stream proteins regulated by FOXP3, the AF and ANC cells were analyzed using a label-free quantitative proteomic workflow in five biological replicates (Fig. 2). After FASP digestion, the resulting tryptic peptides were analyzed using nano-LC-MS/MS with a nano-LC1000 system and an Orbitrap Elite mass spectrometer. The mass spectrometry raw data was processed with Maxquant software, and the results were further filtered with false discovery rate (FDR) of less than 1% at both peptide and protein. As a result, a total of 37,366 unique peptides, corresponding to 3,978 protein groups, were identified (Supplementary Table 1). Among these identified protein groups, 3,313 protein groups, with peptides identified in more than 3 samples, were used for further data analysis (Supplementary Table 2). As shown in Supplementary Table 4, RSD values of the number of MS scans, MS/MS scans, identified MS/MS scans, percentage of identified MS/MS scans, unique peptide sequences, and protein groups across the 10 LC-MS/MS runs were <5%, suggesting the good reproducibility of the LC-MS/MS system.

**Figure 3:**
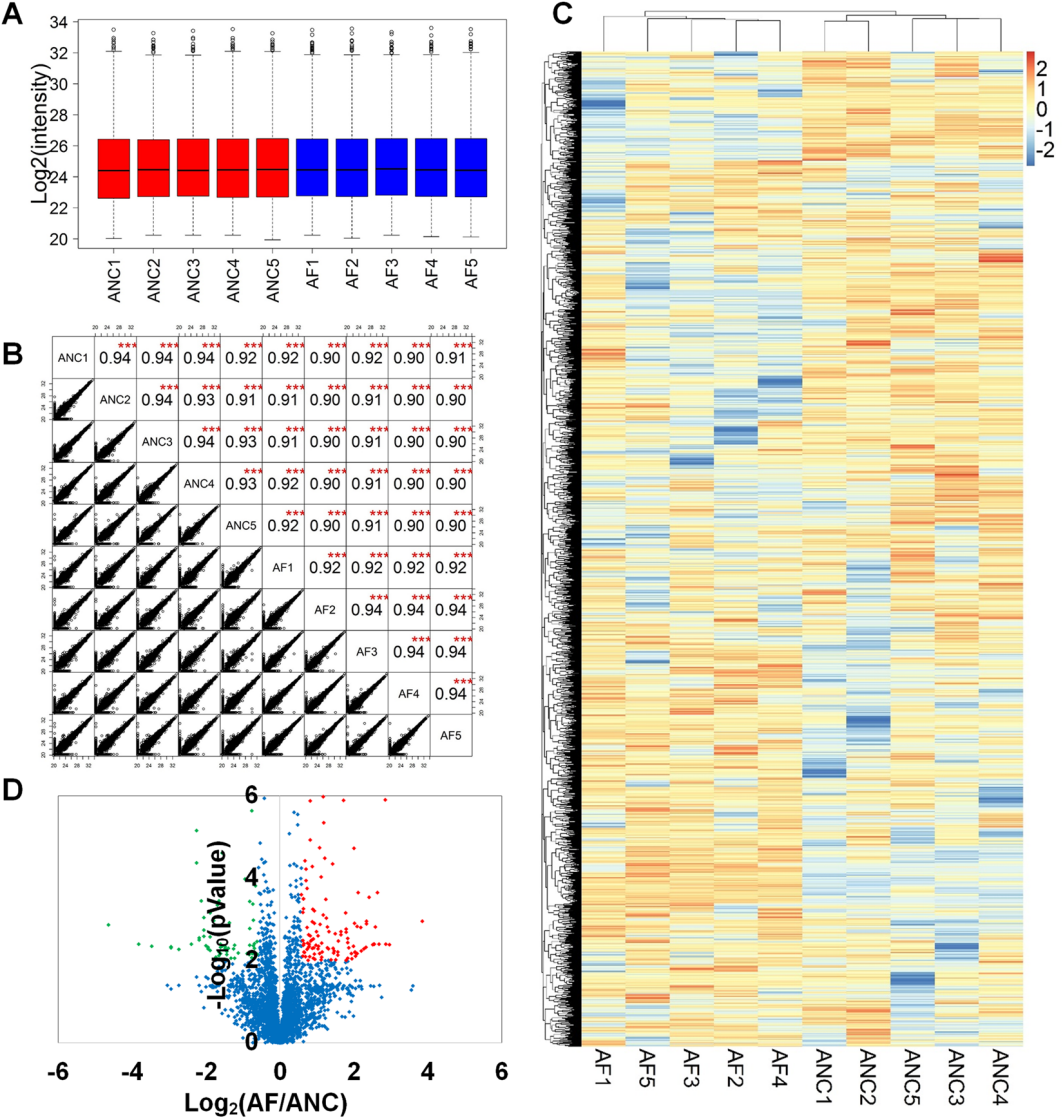
Quantitative profiling of AF cells and ANC cells. (**A**) Box plot of the protein LFQ intensity (Log_2_) for each sample. (**B**) A matrix of scatter plots and Pearson correlation coefficient of LFQ intensities between LC−MS/MS runs. (**C**) Heatmap for HCA analysis. Clustering was performed on the LFQ intensity of total 3,313 proteins. Blue and brick red blocks, respectively, represent low and high expression of proteins relative to the average, whereas white blocks indicate no difference in expression. (**D**) Volcano plot showing P values (−Log_10_) versus protein ratio of AF/ANC cells (Log_2_) of all 3,313 proteins fulfilling strict quantitation criteria. (Red, 119 up-regulated proteins; green, 67 down-regulated proteins; blue, not significantly changed between AF and ANC cells; t-test P value < = 0.01 and foldchange > = 1.5).

Box plot analysis was applied to comparing the Log_2_ transformed LFQ intensity of the AF and ANC cell samples. The median of Log_2_(LFQ) intensity was almost at the same level across the 10 samples, which indicated that the results of the LFQ analysis had no biases toward different samples (Fig. 3A). Relative LFQ profiling was highly reproducible between the biological replicates of the same cell lines and between the LC-MS/MS runs from different cell lines. The correlation coefficient of the Log_2_(LFQ) intensities from each two different samples was greater than 0.90, indicating that the performance of LC-MS/MS system is robust and reproducible (Fig. 3B).

In addition, a heat map plot of a hierarchical clustering analysis (HCA) of the total protein intensities from 10 samples was generated to globally discern the proteomic changes (Fig. 3C). As indicated in the “tree” diagrams of the cluster results for column, 5 samples of the AF cell lines and 5 samples of the ANC cell lines were intriguingly classified into two respective clusters, indicating the profound proteomic regulation between two cell lines. Moreover, the protein expression pattern within each cluster was very similar. Overall, our data showed excellent consistency and reproducibility, could gain a system-wide map of FOXP3 induced protein expression and shed new light on the mechanism of FOXP3 in gastric cancer.

The high quality quantification data thus allowed an accurate comparison between the AF and ANC cells. A strict criterion including protein fold change >= 1.5 and p value of t-test analysis <= 0.01 was used for significant protein screening. Collectively, 186 proteins were significantly changed between AF and ANC cells; among them, 67 proteins were down-regulated and 119 proteins were up-regulated in the AF cells (Fig. 3D). The normalized quantification data from these 186 proteins are shown in Supplementary Table 3.

### Protein-protein Interaction Network Analysis Using 186 significantly Changed Proteins with or without FOXP3 overexpression

**Figure 4:**
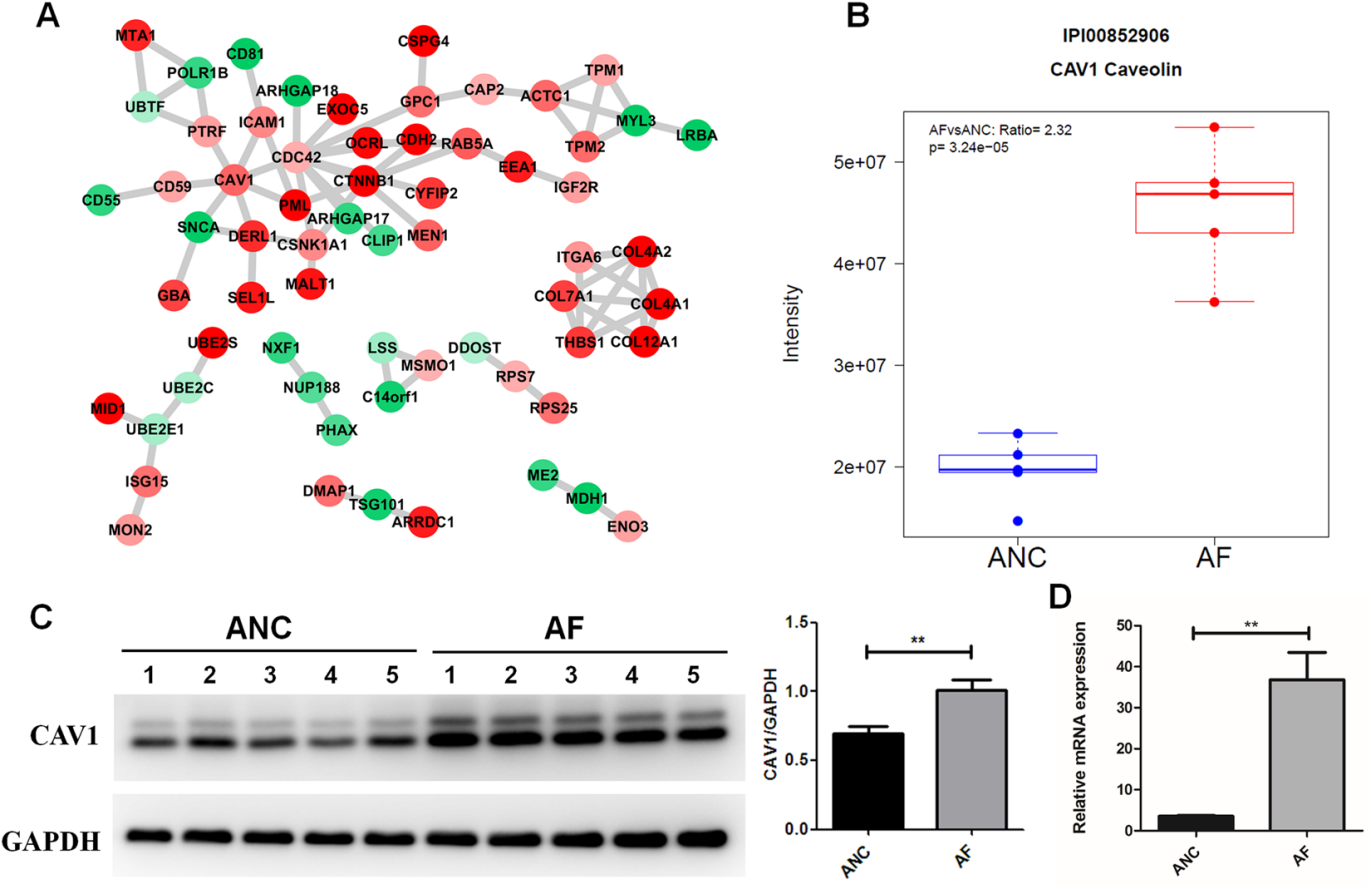
CAV1 was up-regulated in AF cells. (**A**)Protein−protein interaction network of significantly changed proteins. (**B**) Scatter and box plots for the LFQ intensity of CAV1 quantified by label-free proteomics. Western blot analysis (**C**) and qRT-PCR (**D**) both confirmed the up-regulation of CAV1 in FOXP3-overexpressing cells.

Molecular processes within a cell tend to be carried out by molecular machines that are built from a large number of protein components organized by their protein-protein interactions. Thus, a full understanding of FOXP3 induced phenotype in Gastric cells will require mechanism descriptions of how protein interaction networks are perturbed. We uploaded the 186 significant proteins into the STRING database to generate a protein-protein interaction network (Fig. 4A). The PPI network composed of 64 proteins connected to each other, including 21 down-regulated proteins and 43 up-regulated proteins in AF cells. The proteins with direct protein-protein interaction tended to have the same regulation direction, either up-regulated or down-regulated. Based on topology structure of PPI networks, CAV1 functionated as a hub protein (six proteins connect directly with CAV1, five of which up-regulated and only one down-regulated), indicating that it may play a central role in FOXP3 induced cellular function. In addition, KEGG pathways enrichment analysis also revealed that focal adhesion pathway, in which CAV1 serves as an adaptor molecule, was overrepresented in the significantly changed proteins (Supplementary Fig. 1). As a major structural component protein of caveolae^29,30^, CAV1 is implicated in regulating multiple cancer-associated processes, ranging from cellular transformation, tumor growth, invasion and metastasis, to multidrug resistance and angiogenesis^31^. Loss of epithelial CAV1 may promote malignant progression and low CAFs CAV1 level herald worse outcome of GC patient^32^, indicating that CAV1 may play an important role as the down-stream effector of FOXP3 in regulation the adhesion, migration and invasion capabilities of GC cells.

Caveolae is a specialized form of membrane lipid raft, characterized by flask-shaped cavities in the cell membrane^33,34^. CAV1 is the principal scaffolding protein of caveolae; it interacts directly with signaling molecules (receptors, kinases, adhesion molecules, and G-proteins) and controls their subcellular distribution and activation status. This function of CAV1 explains its effects on multiple cellular processes, such as molecular transport, cell proliferation, adhesion, migration and signal transductions^35–37^. Recent studies reported that the loss of CAV1 in the tumor stroma results in an activated tumor microenvironment and was significantly related to metastasis, early tumor recurrence, and poor clinical outcome in breast cancer, prostate cancer, and other solid tumors^32,38–41^. Simultaneously, high CAV1 expression could predict a better overall survival in gastric cancer in a meta-analysis^42^.

Several studies have suggested that CAV1 played a tumor-suppressor role *in vitro* and *in vivo* ^43,44^. Others reported that CAV1 increased in the more advanced stages of cancer^45,46^. A recent study showed that CAV1 levels were markedly decreased in the GC samples as compared with the tumor-free tissues. In human GC cell lines, the expression of CAV1 is low in cells originating from primary tumors (AGS and SNU-1), but it is high in cell lines derived from distant metastases (MKN-7 and MKN-45). Ectopic CAV1 could slow proliferation of adherent epithelial AGS cells, while in metastatic MKN-45 cells, loss of CAV1 increased tumor growth, indicating that CAV1 acts as a tumor suppressor at the early stages of carcinogenesis. However, at later stages of tumor progression, increased secretion of CAV1 by tumor and stromal cells could induce tumor invasion and metastasis^47^. Therefore, CAV1 exhibits a stage-dependent biphasic pattern in tumorigenesis.

Cavin1 (also known as PTRF for Polymerase I and transcript release factor) which is an important structural element of caveolae was also detected in our proteomic analysis with an AF/ANC ratio of 1.68 and a p-value of 3.87 × 10^−3^. Down-regulation of Cavin1 induced cell migration and contributed significantly to tumor progression and metastasis in prostate cancer cells^48,49^. Cavin1 and CAV1 are co-transcriptionally regulated, a loss of Cavin1 will be associated with a loss of CAV1 as well. Cavin1 was shown to stabilize CAV1 expression or activity by inhibiting its internalization and subsequent lysosomal degradation in pancreatic cancer^50^. Similarly, in breast cancer, the Cavin1 expression is down-regulated as a result of promoter methylation. As CAV1 and Cavin1 are both required for caveolae assembly and function, the tumor suppression function of CAV1 may be due to the deregulation of caveolae and its down-stream signaling pathways^51^.

Moreover, the dual function of CAV1 in tumor progression can be explained by a recently discovered new role as mechanosensors in the adaptation of cells to mechanical stress^52^. Simply, in primary carcinoma, when proliferative tumor cells are blocked by the basal membrane, functional caveolae is responsive to these forces by flattening out and by releasing free CAV1 in the plasma membrane, which would activate anti-tumoral or inhibit pro-tumoral signaling effectors. At later stages, a series of events including mechanical forces would overwhelm the capacity of caveolae which in turn would activate pro-tumoral or inhibit anti-tumoral signaling effectors^53^.

In our study, we used AGS cells which originated from primary tumors as our cell lines. According to our results, CAV1 were up-regulated in FOXP3-overexpressing cells comparing to control cells, and these results were consistent with the tumor suppresser role of CAV1 in primary tumors as reported.

### Up-regulation of CAV1 by FOXP3-mediated transcription activation

In our proteomics quantitation data CAV1 was up-regulated significantly in the FOXP3-overexpressed cells, with an AF/ANC ratio of 2.32 and a p-value of 3.24 × 10^−5^ (Fig. 4B). Western blot also confirmed that CAV1 was up-regulated (Fig. 4C, Original Western Blot images shown in Supplementary Fig. 5). Thus, we first analyzed the relationship of tumoral FOXP3 expression and CAV1 expression by tissue microarrays containing 90 cases of gastric cancer with a mean of 6 years follow-up time. Prognostic analysis showed that patients with FOXP3-positive tumors had a longer survival time and better prognosis, compared with FOXP3-negative patients (*p* = 0.085), which are consistent with the conclusions of our previous studies. Furthermore, patients with elevated CAV1 expression also displayed a longer survival time and a better prognosis as determined by tissue microarrays (*p* = 0.014) (Supplementary Fig. 2A,B). We further analyzed the correlation of FOXP3 and CAV1 in same gastric cancer samples, revealed that FOXP3 levels are positively correlated with CAV1 levels in gastric cancer (*p* = 0.0001, r = 0.3956) (Supplementary Fig. 2D). We next studied the molecular mechanisms by which the protein level of CAV1 is altered in FOXP3 overexpressed GC cells. As indicated by qPCR, the mRNA level of CAV1 was significantly increased in AF cells (AF/ANC ratio of 10.3 and a p-value < 0.01), Fig. 4D). In addition, after transfecting either control siRNA or FOXP3-targeting siRNA into AGS cells for 48 hours, the FOXP3 siRNAs markedly reduced CAV1 levels (Supplementary Fig. 3).As long as the transcription factor functional role for the FOXP3, we therefore hypothesized that FOXP3 might influence the expression of CAV1 by regulating gene transcription.

**Figure 5:**
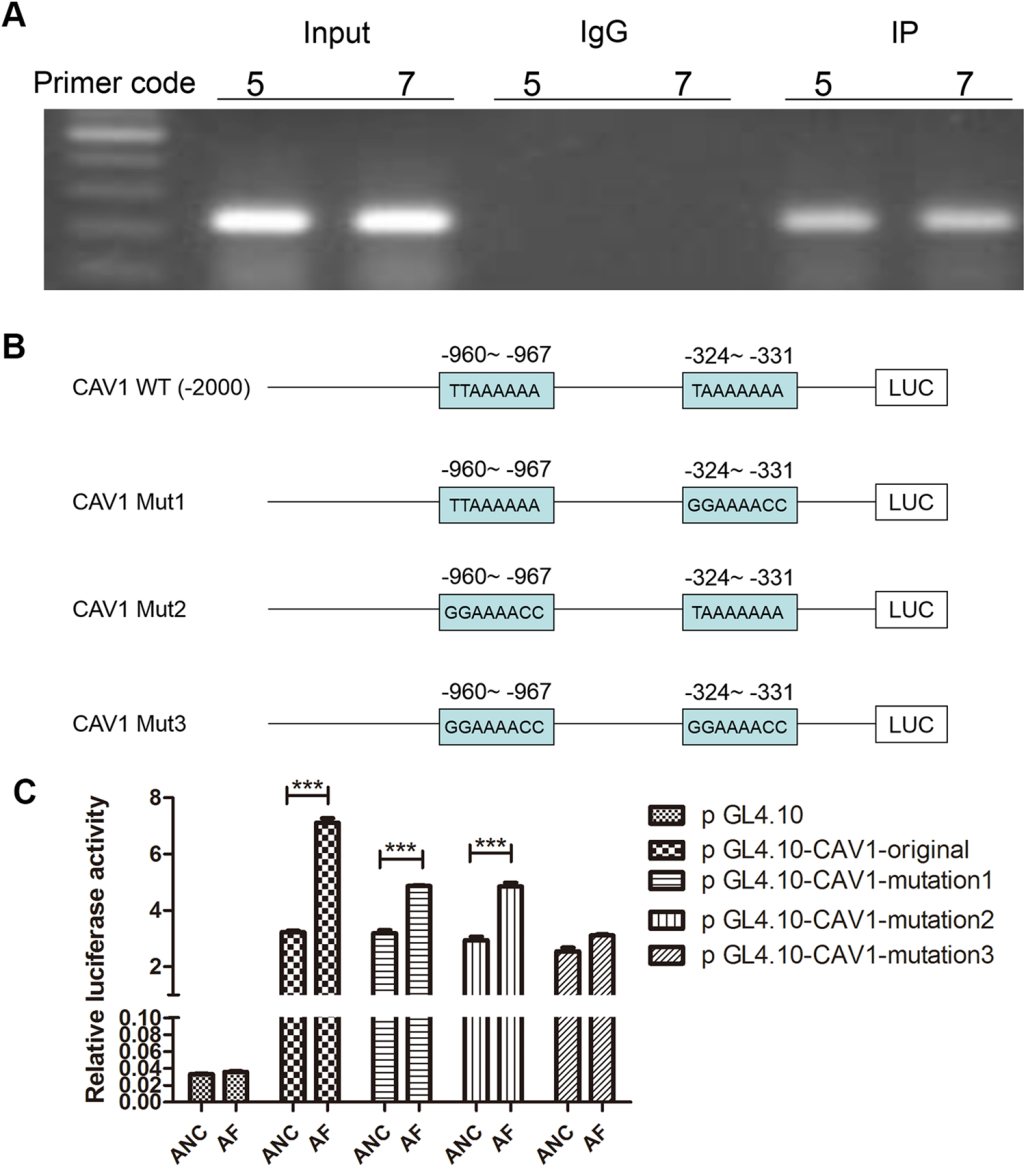
(**A**) ChIP analysis of the recruitment of FOXP3 on the CAV1 promoter. We use IgG as negative controls and Input as blank controls. The DNA fragments covering the 5th and 7th binding sites were detected within the FOXP3 antibody/DNA complex by PCR. (5: −1002 bp~ −751 bp; 7: −501 bp~ −250 bp) (**B**) We constructed a series of luciferase reporter expression vectors: pGL4-CAV1-wild type, pGL4-CAV1-mut1, pGL4-CAV1-mut2, and pGL4-CAV1-mut3. (**C**) FOXP3 as well as its control were cotransfected with CAV1 promoter constructs and internal control pGL-4.10 into the cells. 48 h later, the luciferase activity was measured using luciferase assay substrate. When No.3 mutation site [(−635 ~−967) and (−324 ~331)] was mutated, the activity of promoter significantly changed.

By analyzing the DNA sequence, several consensus binding motif of FOXP3 was found in the 5′ promoter region of CAV1. ChIP-PCR assay was performed to mapping the physical binding location of FOXP3 on −1002 bp to −751 bp and −501 bp to −250 bp from the starting transcription site in the CAV1 gene promoter (Fig. 5A). To test this hypothesis, we constructed a series of luciferase reporter expression vectors (pGL4-CAV1-original), pGL4-CAV1-mut1 (TTAAAAAA to GGAAAACC on −324~−331), pGL4-CAV1-mut2 (TTAAAAAA to GGAAAACC on −960~−967), and pGL4-CAV1-mut3 (TTAAAAAA to GGAAAACC on both −324~−331 and −960~−967) (Fig. 5B), driven by different regions of the CAV1 promoter. Luciferase assays showed that FOXP3 significantly up-regulated CAV1 promoter activity. Moreover, when No.3 mutation site (shown in Fig. 5C) was mutated, the activity of promoter almost completely turned off, suggesting that (−635~−967) and (−324~331) both sites were critical for the FOXP3-mediated regulation of CAV1 promoter activity (Fig. 5C). These results indicated that the expression of CAV1 was regulated by FOXP3 in transcription level, and CAV1 might be a downstream effector molecular of FOXP3 to alter GC cell phenotype.

## Conclusion

In this study, we uncovered that overexpression of FOXP3 significantly decreased migration, invasion and proliferation ability in GC cells. Then we applied global quantitative proteomics to characterize the changes in protein abundance after overexpression of FOXP3 in GC cells. A total of 3978 proteins were identified and quantified, including 186 significantly changed proteins. One of the up-regulated proteins, CAV1 was suggested by protein-protein interaction network-based bioinformatics analysis as well as KEGG pathway to be a candidate downstream molecular of FOXP3. Furthermore, ChIP-PCR and luciferase report assay demonstrated that FOXP3 directly targets CAV1 to positively regulate CAV1 transcription in GC cells originated *in situ*. These results indicate that CAV1 may play an important role as the downstream transcriptional target of FOXP3 in regulation the adhesion, migration and invasion capabilities of GC cells.

## Electronic supplementary material

Supplementary Information

Supplementary Tables 1-4
