# Supplementary material for: Quantitative proteomic Analysis Reveals up-regulation of caveolin-1 in FOXP3-overexpressed human gastric cancer cells
Source: Sci Rep. 2017 Oct 31;7:14460. doi: 10.1038/s41598-017-14453-2 (PMC5663943; doi:10.1038/s41598-017-14453-2)
Supplement: Supplementary file 1 — Supplementary Information [file 41598_2017_14453_MOESM1_ESM.doc]

**Supplementary data for:**

**Quantitative proteomic Analysis Reveals up-regulation of caveolin-1 in FOXP3-overexpressed human gastric cancer cells**

Duyi PAN^1,*^, Jing GAO^3,*^, Xiaoqing ZENG^1,*^,Guifen MA^2^, Na LI^1^, Xiaoquan HUANG^1^, Xuanling DU^1^, Qing MIAO^1^, Jingjing LIAN^1^, Lili XU^1^, Hu ZHOU^3,4#^, Shiyao CHEN^1#^

^1^Department of Gastroenterology, Zhongshan Hospital, Fudan University, Shanghai, China;

^2^Department of Radiotherapy, Zhongshan Hospital, Fudan University, Shanghai, China;

^3^Department of Analytical Chemistry and CAS Key Laboratory of Receptor Research, Shanghai Institute of Materia Medica, Chinese Academy of Sciences, Shanghai, China

^4^University of Chinese Academy of Sciences, Beijing 100049, China

**AUTHOR INFORMATION**

Corresponding Authors

^#^Shiyao Chen.: E-mail: chen.shiyao@zs-hospital.sh.cn.

^#^Hu Zhou: E-mail: [zhouhu@simm.ac.cn](mailto:zhouhu@simm.ac.cn).

^*^Duyi Pan, Jing Gao and Xiaoqing Zeng contributed equally to this work.

**Supplementary Figure Legends**

Supplementary table 1. list of 3978 total identified proteingroups.

Supplementary table 2. Imputated normalized quantification results of 3313 proteins(quantifed in more than 3 samples).

Supplementary table 3. 186 significant proteins with foldchange >=1.5, T-test pvalue <= 0.01.

Supplementary table 4. Summary of maxquant identification results.

Supplementary Figure 1. KEGG pathway mapping of adhesion signaling pathway for significantly changed proteins revealed CAV1 as an adapter protein. The schematic representation showing the adhesion signaling pathway map, which depicts the molecular interaction and reaction network[1-3]. The red color represents the upregulated proteins; light blue corresponds to the downregulated proteins.

Supplementary Figure 2. (A) Kaplan–Meier survival analysis indicates that patients with FoxP3-positive tumors have a longer survival time and better prognosis compared with the patients lacking FoxP3 expression in tumor, as determined by tissue microarrays containing 90 cases of gastric cancer with a mean of 6 years follow-up time (log rank test, P= 0.0855). (B) Patients with high CAV1 level show a longer survival time and better prognosis in tumor tissues (log rank test, P= 0.0141). (C) Strong cytoplasmic staining of both FOXP3 and CAV1 was observed in GC tissues. (D) FoxP3 levels show a positive correlation with CAV1 in tumors (P=0.0001, r= 0.3956).

Supplementary Figure 3. Knockdown of FOXP3 decreased the expression of CAV1. AGS cells were transfected with either three different siRNAs targeting FOXP3 or a control siRNA. After 48 hours, cells were harvested and proteins were analyzed by immunoblotting.

Supplementary Figure 4. Original images of the Western Blots shown in Figure 1B. (A) Original membrane images for Figure 1B. (B) Full membrane from the repeated Western Blot experiment using the same cell lysis samples.

Supplementary Figure 5. Original images of the Western Blots shown in Figure 4C. (A) Original membrane images for Figure 4C. (B) Full membrane from the repeated Western Blot experiment using the same cell lysis samples.

**Supplementary Figure 1**


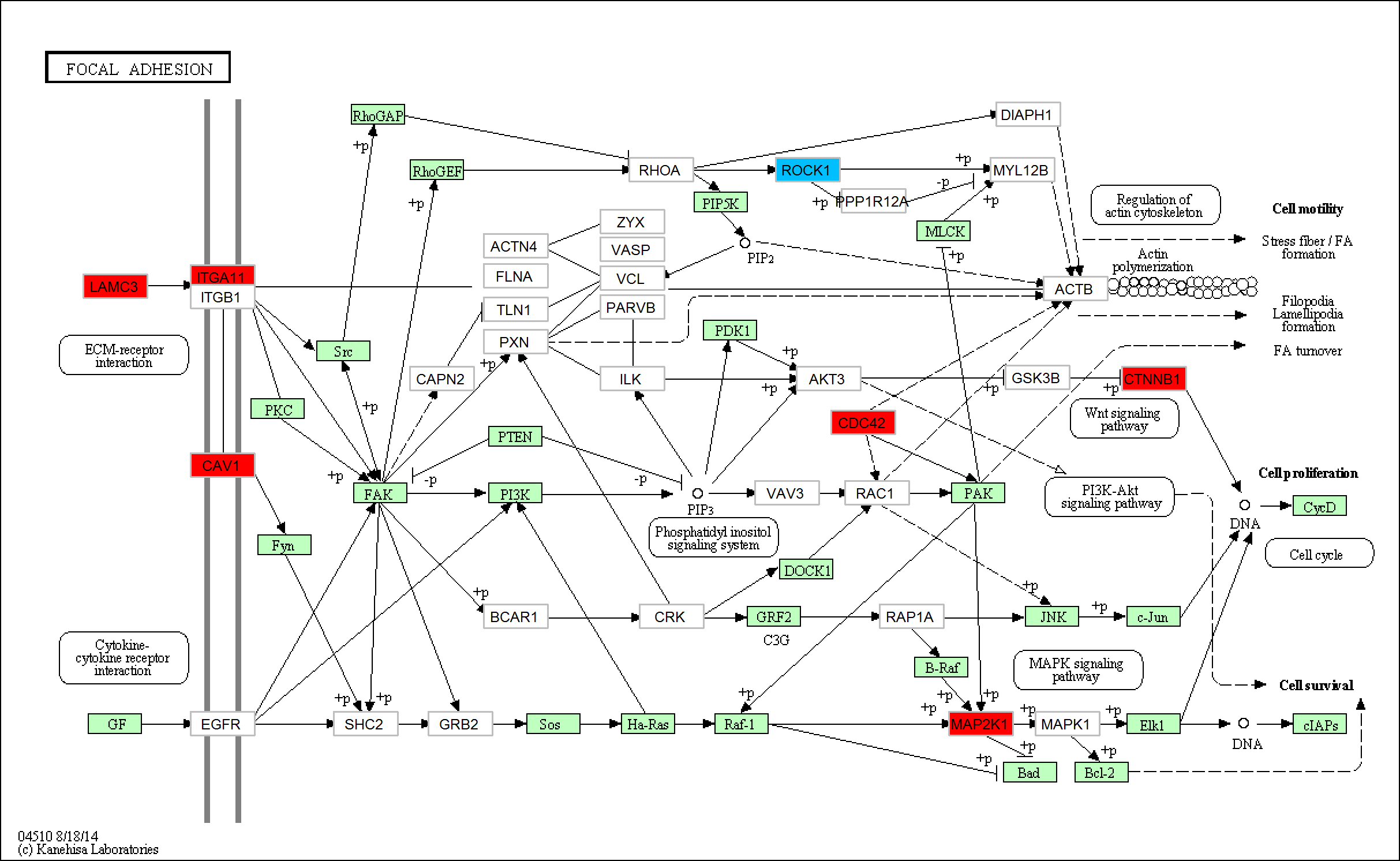


**Supplementary Figure 2**

**
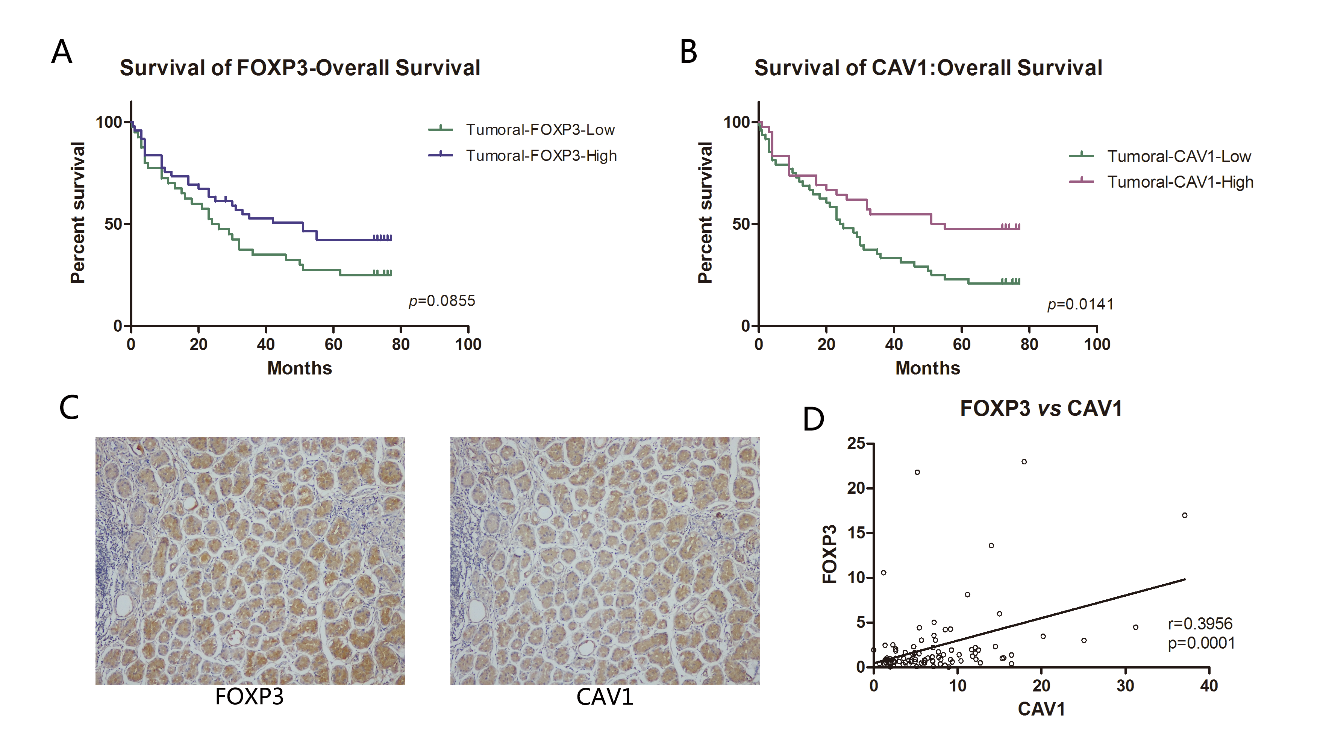
**

**Supplementary Figure 3**


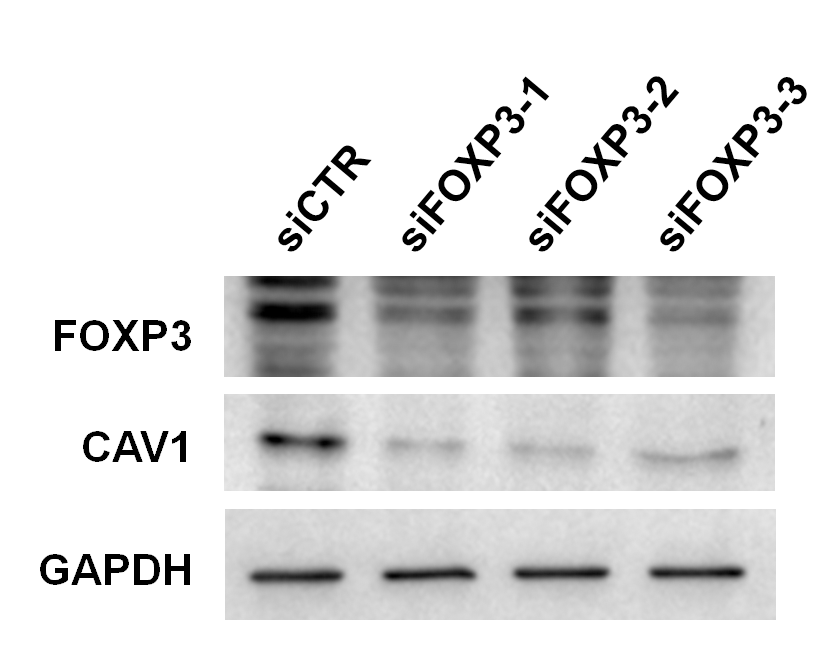


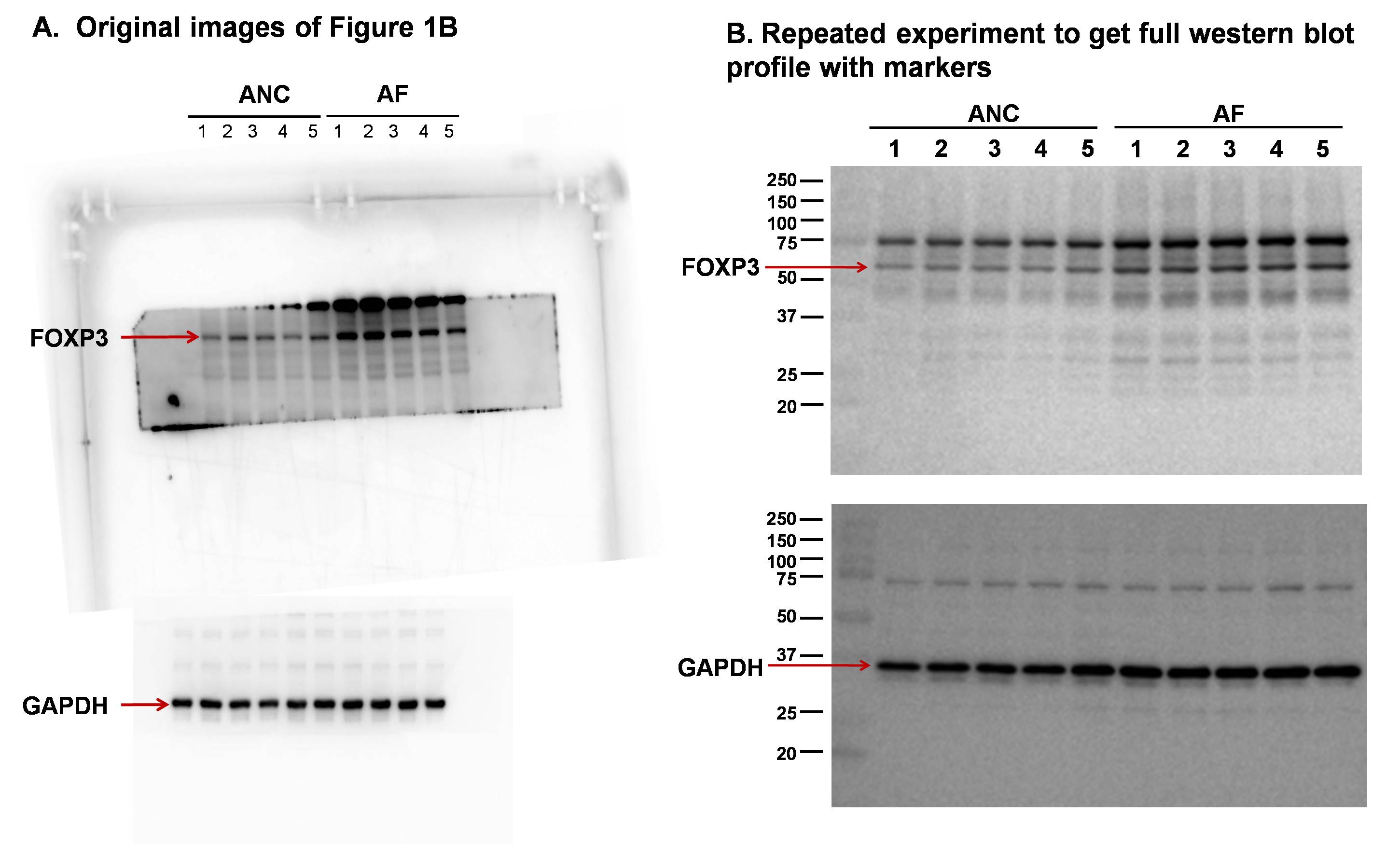


**Supplementary Figure 5**


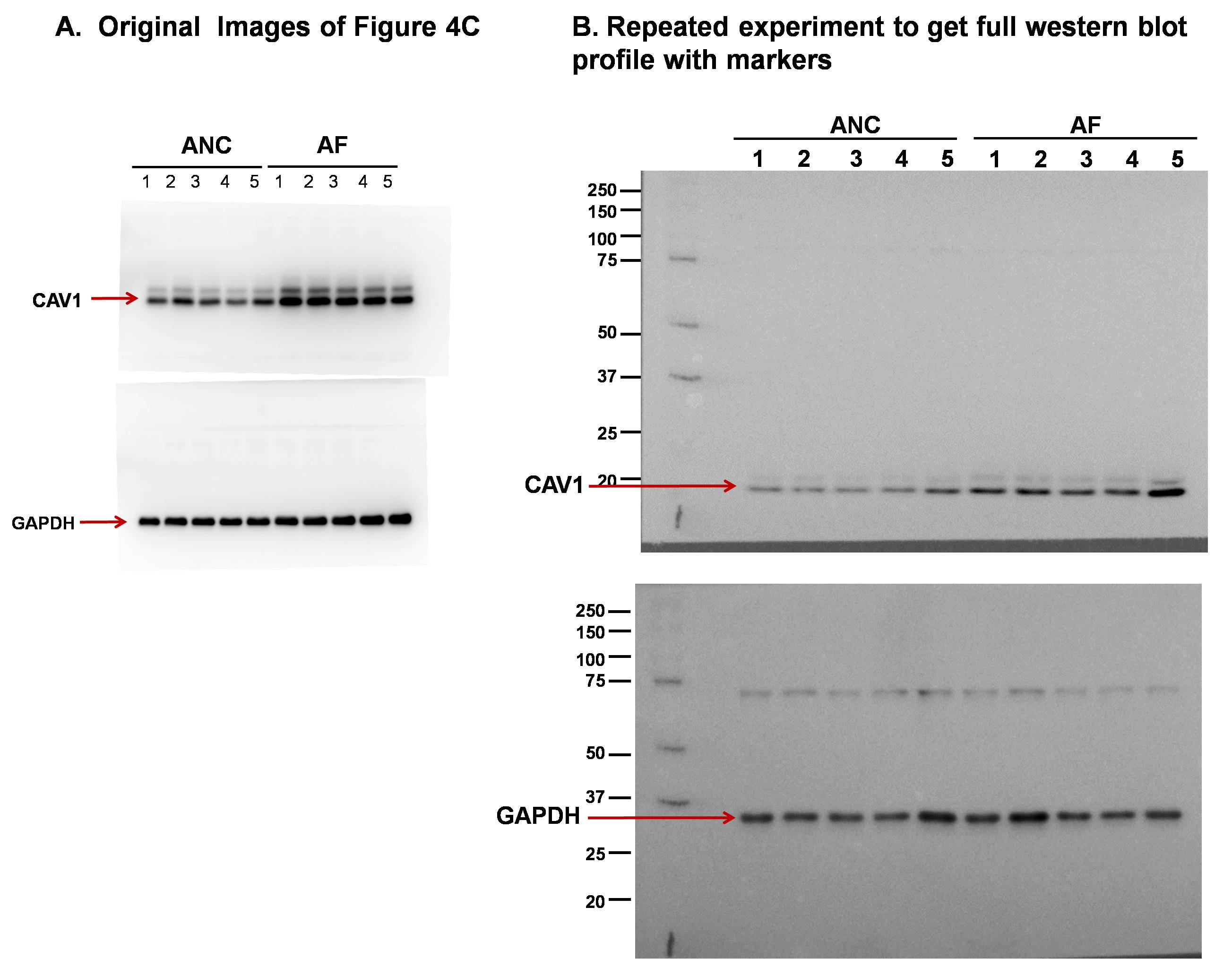


**References:**

1. Kanehisa, M., et al., *KEGG: new perspectives on genomes, pathways, diseases and drugs.* Nucleic Acids Res, 2017. **45**(D1): p. D353-D361.

2. Kanehisa, M. and S. Goto, *KEGG: kyoto encyclopedia of genes and genomes.* Nucleic Acids Res, 2000. **28**(1): p. 27-30.

3. Kanehisa, M., et al., *KEGG as a reference resource for gene and protein annotation.* Nucleic Acids Res, 2016. **44**(D1): p. D457-62.
